# circHECTD1 promotes the silica-induced pulmonary endothelial–mesenchymal transition via HECTD1

**DOI:** 10.1038/s41419-018-0432-1

**Published:** 2018-03-14

**Authors:** Shencun Fang, Huifang Guo, Yusi Cheng, Zewei Zhou, Wei Zhang, Bing Han, Wei Luo, Jing Wang, Weiping Xie, Jie Chao

**Affiliations:** 1 Department of Respiratory Medicine, The First Affiliated Hospital of Nanjing Medical University, 210029 Nanjing, Jiangsu China; 2 Department of Physiology, School of Medicine, Southeast University, 210009 Nanjing, Jiangsu China; 3 Department of Respiration, Zhongda Hospital, School of Medicine, Southeast University, 210009 Nanjing, Jiangsu China; 4 Key Laboratory of Developmental Genes and Human Disease, Southeast University, 210096 Nanjing, China; 5 Department of Pharmacology, School of Medicine, Southeast University, 210009 Nanjing, Jiangsu China

## Abstract

Excessive proliferation and migration of fibroblasts contribute to pulmonary fibrosis in silicosis, and both epithelial cells and endothelial cells participate in the accumulation of fibroblasts via the epithelial–mesenchymal transition (EMT) and the endothelial–mesenchymal transition (EndMT), respectively. A mouse endothelial cell line (MML1) was exposed to silicon dioxide (SiO_2_, 50 μg/cm^2^), and immunofluorescence and western blot analyses were performed to evaluate levels of specific endothelial and mesenchymal markers and to elucidate the mechanisms by which SiO_2_ induces the EndMT. Functional changes were evaluated by analyzing cell migration and proliferation. The mRNA and circular RNA (circRNA) levels were measured using qPCR and fluorescent in situ hybridization (FISH). Lung tissue samples from both Tie2-GFP mice exposed to SiO_2_ and silicosis patients were applied to confirm the observations from in vitro experiments. Based on the results from the current study, SiO_2_ increased the expression of mesenchymal markers (type I collagen (COL1A1), type III collagen (COL3A1) and alpha smooth muscle actin (α-SMA/Acta2)) and decreased the expression of endothelial markers (vascular endothelial cadherin (VE-Cad/Cdh 5) and platelet endothelial cell adhesion molecule-1 (PECAM1)), indicating the occurrence of the EndMT in response to SiO_2_ exposure both in vivo and in vitro. SiO_2_ concomitantly increased circHECTD1 expression, which, in turn, inhibited HECTD1 protein expression. SiO_2_-induced increases in cell proliferation, migration, and changes in marker levels were restored by either a small interfering RNA (siRNA) targeting circHECTD1 or overexpression of HECTD1 via the CRISPR/Cas9 system, confirming the involvement of the circHECTD1/HECTD1 pathway in the EndMT. Moreover, tissue samples from SiO_2_-exposed mice and silicosis patients confirmed the EndMT and change in HECTD1 expression. Our findings reveal a potentially new function for the circHECTD1/HECTD1 pathway and suggest a possible mechanism of fibrosis in patients with pulmonary silicosis.

## Introduction

Silicosis is a pulmonary disease characterized by progressive pulmonary fibrosis caused by long-term inhalation of air containing free silica dust. The excessive proliferation and migration of fibroblasts contributes to pulmonary fibrosis in patients with silicosis^1,2^, and multiple studies have indicated that both epithelial cells and endothelial cells participate in the accumulation of fibroblasts via the epithelial–mesenchymal transition (EMT) and endothelial–mesenchymal transition (EndMT) in different settings^3–5^.

Although mounting evidence has indicated that both the damage to alveolar epithelial cells and subsequent diffuse inflammatory responses are involved in the pathogenesis of pulmonary fibrosis, the EndMT has received little attention in the context of silicosis. The EndMT occurs in in different organs, such as the kidneys^6^, liver, and heart^7^, in patients with fibrotic disorders, as well as in patients with diabetes^8^, and metastatic tumors^7^. The EndMT is characterized by the loss of endothelial-specific markers, the acquisition of the mesenchymal or myofibroblast phenotype and the expression of mesenchymal cell products, such as α-smooth muscle actin (α-SMA) and type I collagen (Col I/COL1A1)^9^.

Noncoding RNAs are involved in the EndMT in different diseases, although the detailed mechanisms remain unclear^10–12^. Circular RNAs (circRNAs), which are produced by reverse splicing, comprise a new class of noncoding RNAs and have become a hot topic of research in recent years^13^. circRNAs not only affect mRNA transcriptional levels in the nucleus but also adsorb miRNAs in the cytoplasm or directly interact with specific proteins to affect their transcriptional or post-transcriptional levels^13,14^. For example, the circRNA ciRS-7 acts as a sponge for miR-7, and ciRS-7 is resistant to miRNA-mediated target destabilization, thus strongly suppressing miR-7 activity^15^. The circRNA HIPK2 functions as an endogenous microRNA-124 sponge to increase sigma non-opioid intracellular receptor 1 expression^16^. In addition, circRNAs also affect gene transcription through their associations with phosphorylated Pol II^17^, and circRNAs can compete with the pre-mRNA splicing machinery^18^. A recent study from our laboratory based on a circRNA microarray analysis identified 120 circRNAs in the lung that were differentially expressed in silicon dioxide (SiO_2_)-treated mice compared to normal mice, indicating the fundamental roles of circRNAs in pathological processes induced by SiO_2_.

In the current study, both circHECTD1 and HECTD1 were involved in the SiO_2_-induced EndMT by promoting endothelial cell migration and activation. These findings reveal a novel function for circRNAs in SiO_2_-induced fibrosis and suggest that the circHECTD1/HECTD1 pathway may be involved in multiple steps of the fibrosis process.

## Materials and methods

### Reagents

SiO_2_, 80% of which had a particle diameter of less than 5 μm, was purchased from Sigma (S5631), selected via sedimentation according to Stokes’ law, acid hydrolyzed, and baked overnight (200 °C for 16 h)^19^. The Col I/COL1A1 (BS1530) and type III collagen (Col III/COL3A1, BS1531) antibodies were purchased from BioWord®. The α-SMA/Acta2 antibody (14395-1-AP) was purchased from Proteintech®. The HECTD1 (SC-134976), vascular endothelial cadherin (VE-Cad/Cdh 5, SC-9989), and platelet endothelial cell adhesion molecule-1 (PECAM 1/CD31, SC-1506) antibodies were purchased from Santa Cruz Biotechnology.

### Animals

STOCK TEK-GFP 287 Sato/JNju (Tie2-GFP) mice (aged 6–8 weeks, 17–20 g) were obtained from the Laboratory Animal Center of Nanjing Medical University (Nanjing China), and GFP was only expressed in endothelial cells. All animals were males and housed (4 per cage) in a temperature-controlled room (25 °C, 50% relative humidity) with a 12-h light/dark cycle. All animal procedures were performed in strict accordance with the ARRIVE guidelines, and the animal protocols were approved by the Institutional Animal Care and Use Committee of Southeast University.

### Cell culture

Mouse microvascular lung (MML1) cells were purchased from Feibo® and maintained in T25 flasks in Dulbecco’s Modified Eagle’s Medium (DMEM) containing 10% fetal bovine serum (FBS). MML1 cells were stored in liquid nitrogen between passages 3–10 (P3-10). A vial of MML-1 cells was thawed, plated, and passaged until confluence for each experiment, which was performed between passages P5 and P10. Human umbilical vein endothelial cells (HUVECs) were purchased from ScienCell® and maintained in T25 flasks in DMEM supplemented with 10% FBS. HUVECs from passages 3–7 (P3-7) were stored in liquid nitrogen. A vial of P3-7 HUVECs was thawed, plated, and passaged upon reaching confluence to perform each experiment, and each experiment was performed using HUVECs between P10 and P15^20^.

### Establishment of a mouse model of silicosis

Animals were anesthetized with an intraperitoneal injection of pentobarbital sodium, and their tracheae were surgically exposed. A prepared SiO_2_ suspension (0.2 g/kg in 50 mg/mL saline) was instilled intratracheally in one dose. Lung tissues were collected 28 days after treatment. Control animals were administered the same volume of sterile saline, as previously described^21^.

### 3-(4,5-Dimethylthiazol-2-yl)-2,5-diphenyltetrazolium bromide (MTT) assay

Cell viability was measured using 3-(4,5-dimethylthiazol-2-yl)-2,5-diphenyltetrazolium bromide (MTT) assays^1^. Briefly, cells were collected and seeded in 96-well plates at a density of 10^4^ cells/well; the wells on the edges of each 96-well plate were filled with PBS. Then, cells were incubated in a 37 °C incubator with a 5% CO_2_ atmosphere for 24 h and incubated with 50 μg/cm^2^ SiO_2_ for 0, 12, 24, 48, and 72 h. Twenty microliters of MTT dissolved in Hank’s balanced salt solution were added to each well, and the plates were incubated in a 5% CO_2_ incubator for 1–4 h. Finally, after removing the cell supernatant, cells were treated with 200 μL of dimethyl sulfoxide and agitated on a shaker for 10 min to fully dissolve the formazan crystals. A BioTek microplate reader (SYNERGYH1; BioTek, Highland Park, VT, USA) was used to measure the absorbance of each well at a wavelength of 570 nm. Each experiment was repeated at least three times.

### Western blotting

Cells were cultured in 24-well plates in an incubator containing 5% CO_2_ at 37 °C, and after a variable period of time, cells were washed three times with cold PBS and lysed using a mammalian cell lysis kit (MCL1-1KT, Sigma-Aldrich), according to the manufacturer’s instructions. Equal concentrations of proteins were separated by 8% SDS-PAGE gel electrophoresis under reducing conditions and transferred to PVDF membranes. The PVDF membranes were then blocked with 5% nonfat dry milk in TBST and mildly agitated on a shaker for 1 h at room temperature, followed by incubations with the indicated primary antibodies overnight at 4 °C. Membranes were washed three times with TBS/0.1% Tween 20 for 8 min per wash and incubated with an alkaline phosphatase-conjugated secondary antibody (1:5000 dilution) in 5% nonfat dry milk for 60 min at room temperature. Finally, the PVDF membranes were washed, and the proteins were detected by densitometry using ImageJ software (NIH). Each western blot was repeated at least three times^1^.

### In vitro scratch assay

An in vitro scratch assay was performed to evaluate cell migration in a 2D culture system. Briefly, 1 × 10^5^ MML1 cells were seeded in 24-well tissue culture plates and cultured in growth medium in a 37 °C incubator in a 5% CO_2_ atmosphere until they reached approximately 70–80% confluence. Then, a sterile 200-µL pipette tip was used to gently scratch the cell monolayer to generate a straight line with a suitable width. Similarly, a second straight line was lightly scratched perpendicular to the first line to create a cross-shaped cellular gap in each well of the 24-well tissue culture plate. Each well was washed twice with fresh growth medium to remove the cell debris, and fresh medium was then added to each well to enable normal cell growth. Simultaneously, each well was incubated with 50 μg/cm^2^ SiO_2_, and digital images of the scratches were captured at 0, 12, 36, and 48 h. The ImageJ software was used to quantitatively evaluate the widths of the cell gaps. Each well was examined at least three times^1^.

### Quantitative real-time PCR

The primers are listed in Table S1. TRIzol reagent (Invitrogen) was used to extract the RNA according to the manufacturer’s instructions. Total RNA (mRNA/circRNA) was reverse transcribed using iScript cDNA synthesis kits (Bio-Rad) according to the manufacturer’s instructions. An SsoFast EvaGreen Supermix RT-PCR kit (Bio-Rad) was used to conduct quantitative RT-PCR assays, and the amount of the target RNA was normalized to an endogenous reference (GAPDH) in each experiment. Cycle threshold (Ct) and ΔCT values were analyzed after standardizing the target RNA to the GAPDH RNA. The ΔΔCT quantification method was performed using the Opticon Monitor software (Bio-Rad) to compare the relative expression levels of the treated MML1 cells to the control MML1 cells. The RQ values were determined by assessing the relative fold changes. Quantitative real-time PCR primers were designed using online software and synthesized by GeneChem. Each quantitative real-time PCR analysis was repeated at least three times^16^.

### Immunocytochemistry

Treated cells were washed twice with PBS and fixed with 4% paraformaldehyde in PBS overnight at 4 °C. Then, after two washes, the coverslips were incubated with 0.3% Triton X-100 in PBS at room temperature for 15 min. The permeabilized samples were blocked with 10% NGS in 0.3% Triton X-100 at room temperature for 2 h. Primary antibodies were diluted in PBS containing 10% NGS and 0.3% Triton X-100, and the blocked samples were incubated in primary antibodies overnight at 4 °C. Samples were washed three times and incubated with secondary antibodies at room temperature for 2 h. A mounting solution (Prolong® Gold antifade reagent with DAPI; P36931, Life Technologies) was used to mount the samples after three washes with PBS. Images of the cells were captured using a fluorescence microscope until the coverslips dried. Each experiment was repeated at least three times^16^.

### Fluorescent in situ hybridization (FISH)

Cellular circHECTD1 expression was detected using FISH with a mixture of biotin-labeled DNA oligo probes specific for either endogenous or ectopically expressed circHECTD1. Briefly, cells were freshly fixed with 4% paraformaldehyde (PFA) for 15 min at room temperature, washed twice with PBS, immersed in 70% ethanol overnight at 4 °C, permeabilized with 0.25% Triton X-100 for 15 min, and subjected to two 15-min washes with saline-sodium citrate (SSC) buffer. In situ hybridization was performed overnight at 37 °C using 10 pM biotin-labeled DNA oligo probes in hybridization buffer (HB), and this step was followed by serial washes with SSC buffer. The probe sequence is shown in Table S1. Samples were then incubated in blocking buffer (1% BSA and 3% normal goat serum in PBS) for 1 h at room temperature followed by an anti-biotin HRP antibody (1:200) in blocking buffer overnight at 4 °C. Samples were subsequently subjected to 2-min washes with PBS. Finally, the DNA was stained with DAPI, and images of the cells were captured using a fluorescence microscope (Olympus BX53, Olympus America, Inc., Center Valley, PA, USA).

### CRISPR/Cas9 plasmid transfection

The HECTD1 CRISPR activation plasmid (SC-431500-ACT) and control CRISPR activation plasmid (SC-437275) were purchased from Santa Cruz Biotechnology. Approximately 0.5–1 × 10^5^ cells were seeded into 24-well plates (with unparalleled anti-standard medium), and the medium in the 24-well plates was replaced with 200 μL of unparalleled anti-fresh medium per well until the cells reached 40-80% confluence. First, 1.5 μL of the siRNA reagent were added to 10 μL of the transfection medium to form solution A, and the solution was mixed gently at room temperature for 5 min. Second, 0.3 μg of the plasmid was added to 10 μL of the transfection medium to form solution B, and the solution was mixed gently at room temperature for 5 min. Third, solution B was added dropwise to solution A to form solution C, which was immediately vortexed at room temperature and incubated for ≥ 20 min. Finally, solution C was added dropwise to the wells of the 24-well plate, followed by mixing. 12 h after transfection, 1 mL of medium was added to the medium in each well of the 24-well plate, and the medium was discarded and replaced with 1 mL of fresh medium, if the cells were in good condition. The samples were incubated for 24–72 h in a 37 °C incubator containing 5% CO_2_ until use in the western blotting analysis^16^.

### RNA interference targeting circHECTD1 using siRNAs

RNA interference targeting circHECTD1 was performed in MML1 cells using a previously described method^16^, with some modifications. The RNA interference protocol was applied to each well of a 24-well plate. Briefly, 3 μL of the siRNA reagent in serum-free DMEM were added to 10 μL of medium to form solution A, and the solution was mixed gently at room temperature for 5 min. Then, 3 μL of the plasmid in serum-free DMEM were added to 10 μL of medium to form solution B, and the solution was mixed gently at room temperature for 5 min. Then, solution B was added dropwise to solution A to form solution C and incubated at room temperature for ≥20 min. Next, 80 μL of serum-free DMEM were added to solution C to form a 100-µL system solution. Cells were washed with PBS and digested with trypsin, and the appropriate amount of normal medium was added to stop the digestion. Next, 100 μL of a 2 × 10^6^ cells/mL cell suspension was added to the above 100-µL system solution to form a 200-µL system solution. The configured 200-µL system solution was added to the wells of a 24-well plate and incubated in a 37 °C incubator containing 5% CO_2_ for 24–72 h until use in the western blotting analysis.

### Nested matrix model and cell migration assay

A nested collagen matrix model was performed as previously described^22^, with certain modifications. The nested collagen matrix was incubated in an attached state with in DMEM containing 10% FBS for 72 h. The matrix was then removed from the culture well, and 60 µL of a fresh acellular collagen matrix solution (NeoMatrix solution) were added to the center of a new well in a 12-well plate. The newly transferred matrix was covered with 140 µL of the NeoMatrix solution and allowed to polymerize for 1 h at 37 °C in a 5% CO_2_ atmosphere. Then, 2 mL of DMEM containing 10% FBS were added to the well. Cell migration out of the nested matrix and into the acellular NeoMatrix was quantified by fluorescence microscopy after 24 h of nesting. Digital images (constant dimensions of 1000 × 800 µm) were captured using an EVOS® FL Cell Imaging microscope (Life Technologies, Grand Island, NY, USA) from 3 to 5 randomly selected microscopic fields at the interface of the nested matrix and acellular NeoMatrix. Migration of MML1 cells was quantified by counting the number of cells that had clearly migrated out of the nested matrix into the cell-free matrix. The maximum migration distance was quantified by identifying the cells that had migrated the greatest distance into the cell-free matrix. The number of cells that had migrated out of the nested matrix per field was also quantified from the digital micrographs.

### Sirius scarlet stains

For the histological analysis of pulmonary fibrosis, the lung was extracted, fixed with 4% formalin and dehydrated in a 30% sucrose solution. Sections of the lung were examined using a Sirius red staining kit (ab150681, Abcam), according to the manufacturer’ s instructions^21^.

### Statistics

Data are presented as the means ± standard errors of the means. Unpaired numerical data were compared using an unpaired *t*-test (two groups) or analysis of variance (more than two groups). A *P*-value <0.05 was regarded as statistically significant.

## Results

### SiO_2_ induces the EndMT

**Fig. 1:**
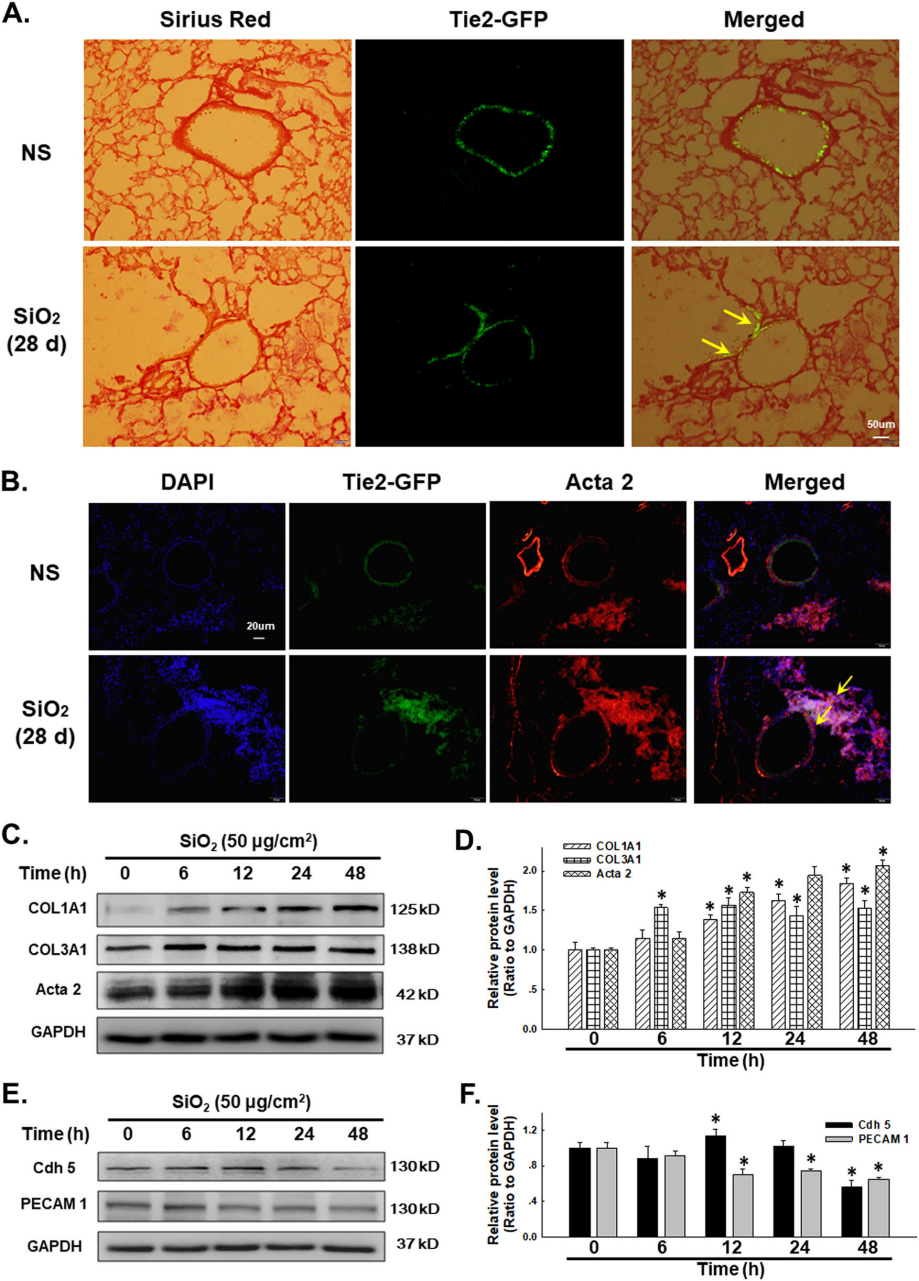
SiO_2_ exposure induces the EndMT in vivo and in vitro. **a** Representative images of Sirius red staining in lung sections show the amount of Sirius red-stained collagen associated that co-localized with Tie2-GFP in mice treated with SiO_2_. **b** Representative images of immunohistochemical staining show the co-localization of TEK-GFP with α-SMA/Acta2 in TEK-GFP 287 Sato/JNju mice treated with SiO_2_, indicating that SiO_2_ induced the EndMT. **c** Representative western blot showing the effects of SiO_2_ (50 μg/cm^2^) on levels of the mesenchymal markers Col I/COL1A1, Col III/COL3A1 and α-SMA/Acta2. **d** Densitometric analyses of five separate experiments show that SiO_2_ increases Col I/COL1A1, Col III/COL3A1 and α-SMA/Acta2 expression in a time-dependent manner. *P < 0.05 vs. the expression of the corresponding protein at 0 h. **e** Representative western blot showing the effects of SiO_2_ (50 μg/cm^2^) on levels of the endothelial markers VE-Cad/Cdh-5 and PECAM 1/CD31. **f** Densitometric analyses of five separate experiments show that SiO_2_ decreases VE-Cad/Cdh-5 and PECAM 1/CD31 expression in a time-dependent manner. *P < 0.05 vs. corresponding protein at 0 h

**Fig. 2:**
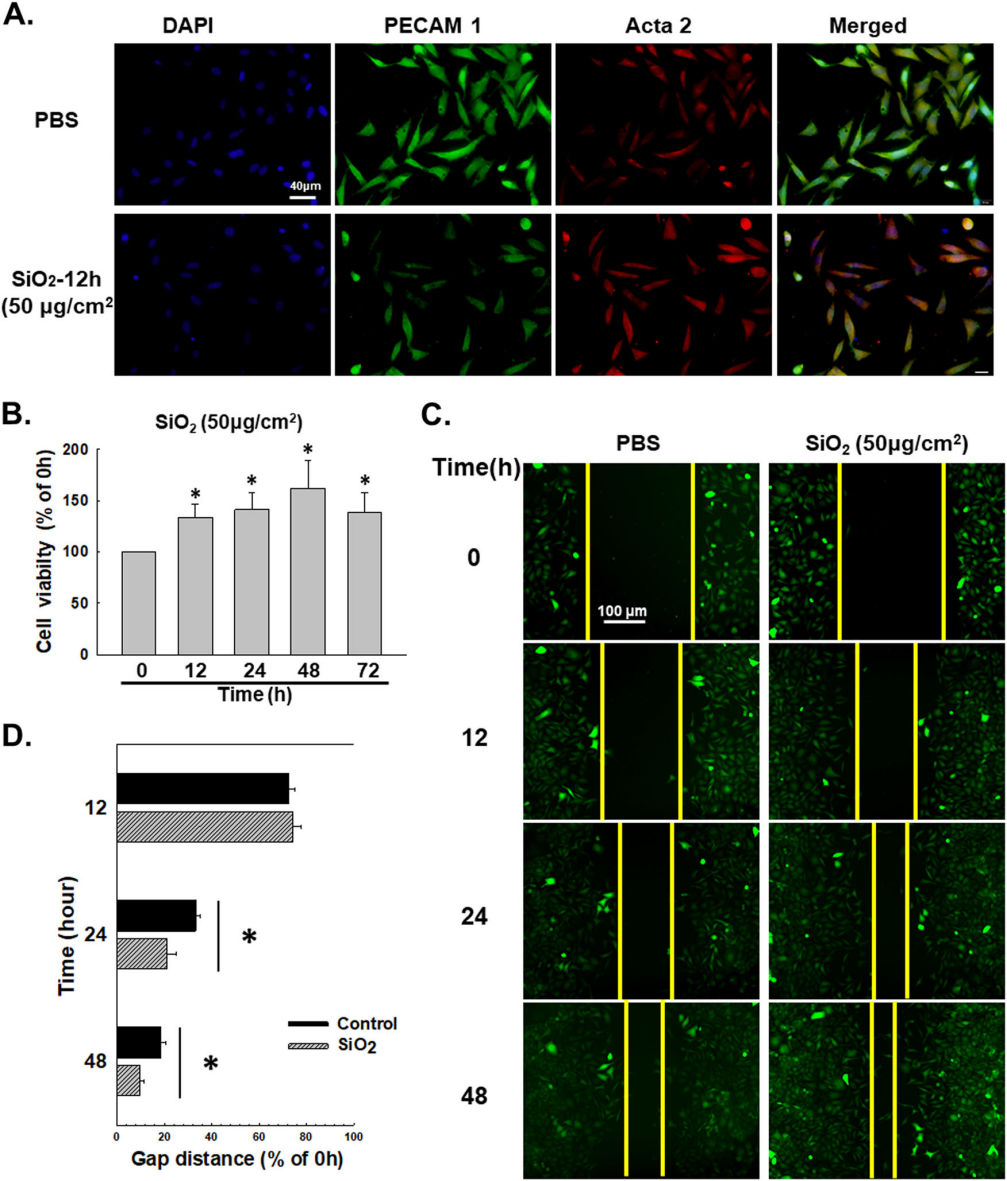
SiO_2_ induces the proliferation and migration of MML1 cells. **a** Representative images of immunocytochemical staining show that SiO_2_ (50 μg/cm^2^) decreases PECAM 1/CD31 expression and increases α-SMA/Acta2 expression in MML1 cells. **b** MTT assay showing the SiO_2_-induced increase in the viability of MML1 cells. *P < 0.05 vs. the 0-h group, *n* = 5. **c** Representative images showing the effects of SiO_2_ on GFP-labeled MML1 cell migration in scratch assays. Scale bar = 80 µm. **d** Quantification of the scratch gap distances in six separate experiments. *P < 0.05 vs. the control group at the corresponding time point

According to a previous study from our laboratory, SiO_2_ induces the EndMT in HUVECs^1^. However, researchers have not clearly determined whether SiO_2_ also induces the EndMT in vivo. In the current study, Tie2-GFP mice were analyzed to determine whether SiO_2_ induced an EndMT response in the lungs of these mice using the Sirius red staining assay, which enabled differential measurements of the amount of collagen deposited in endothelial cells. The representative images of Sirius red staining in lung sections shown in Fig. 1a,  S1a–b show the amount of Sirius red-stained collagen that co-localized with GFP after the SiO_2_ treatment. Meanwhile, as shown in Fig. 1b, GFP co-localized with α-SMA/Acta2, indicating that SiO_2_ induced the EndMT. MML1 was used to confirm the in vivo observations. As shown in Fig. 1c, d, SiO_2_ induced a rapid and sustained increase in Col I/COL1A1, Col III/COL3A1 and α-SMA/Acta2 levels, which are specific mesenchymal markers, indicating the transformation from the endothelial phenotype; however, levels of VE-Cad/Cdh-5 and PECAM 1/CD31, which are specific endothelial markers, showed a delayed and sustained decrease in response to the SiO_2_ treatment (Fig. 2e, f). The changes in both the endothelial and mesenchymal markers were confirmed by immunostaining (Fig. 2a).

### SiO_2_ induces MML1 cell proliferation and migration

Based on accumulating evidence, changes in cell proliferation and migration comprise the onset of the EndMT and pulmonary fibrosis^23–26^. Cell migration and viability were evaluated following exposure to SiO_2_ to further determine the functional effects of endothelial marker loss and mesenchymal marker acquisition by these cells. As shown in Fig. 2b, SiO_2_ exposure increased cell viability. Moreover, cell migration began to increase after 24 h of SiO_2_ exposure (Fig. 2c, d), indicating that functional changes were associated with changes in the endothelial and mesenchymal markers.

### SiO_2_ induces circHECTD1 expression in MML1 cells

**Fig. 3:**
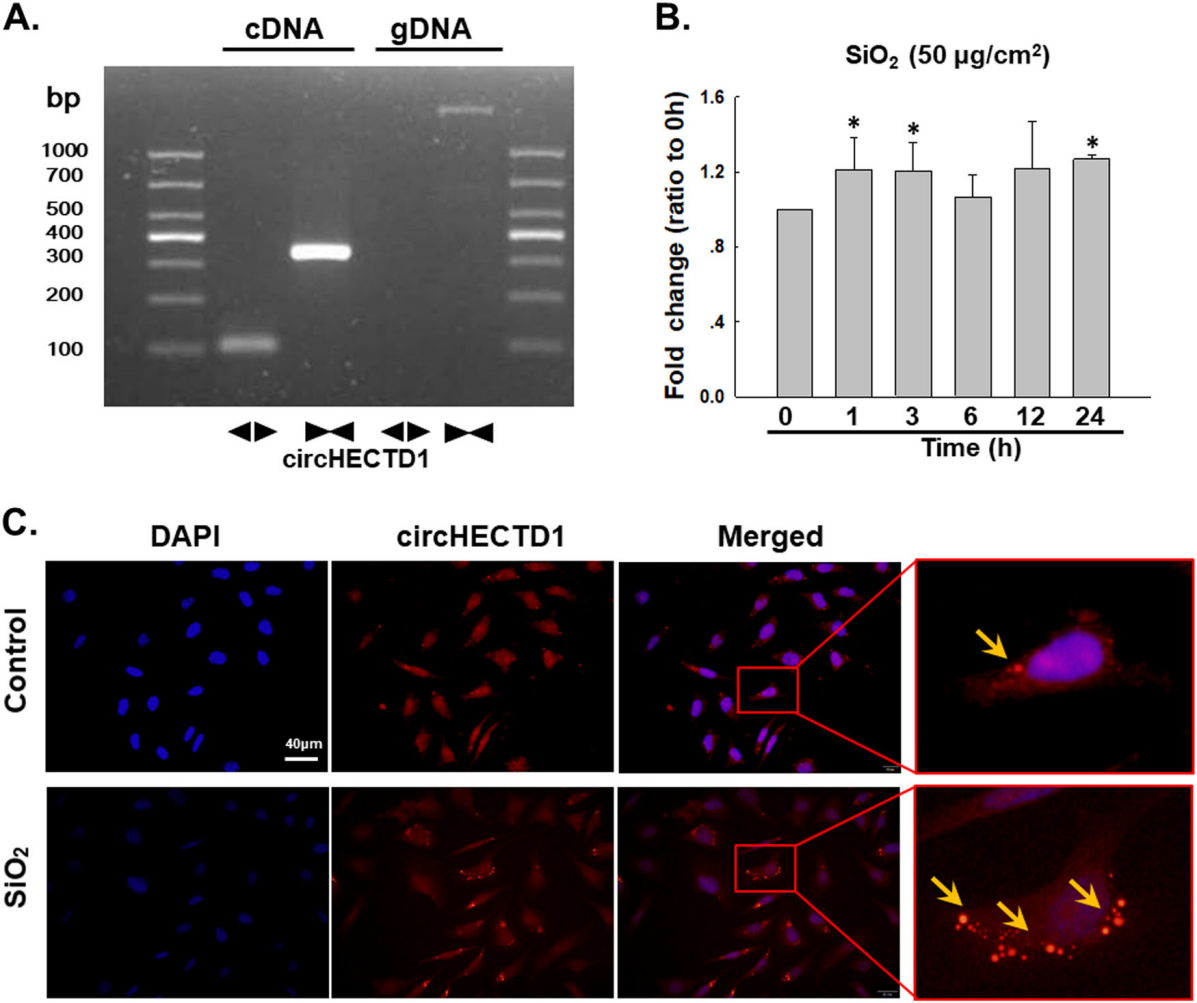
SiO_2_ induces circHECTD1 expression in MML1 cells. **a** Divergent primers amplified circRNAs from cDNAs, but not from gDNA. **b** As shown in the qRT-PCR analysis, circHECTD1 expression increased in the MML1 cells exposed to SiO_2_ (*n* = 5). *P < 0.05 vs. circHECTD1 expression at 0 h. **c** FISH analysis showing that circHECTD1 expression increases in MML1 cells exposed to SiO_2_. circHECTD1 was labeled with fluorescein isothiocyanate

Based on recent reports, circRNAs are involved in different diseases^27–31^, but researchers have not clearly determined whether circRNAs are involved in the development of silicosis. A recent circRNA microarray analysis-based study revealed 120 differentially expressed circRNAs in the lungs of silicosis mice (Figure S2), among which circHECTD1 is of particular interest because its host gene, HECTD1, a candidate E3 ubiquitin ligase, may be involved in SiO_2_-induced fibrosis by inducing ubiquitination^32^. Based on previous data from our laboratory, ZC3H12A/MCPIP1 may mediate macrophage activation and fibroblast proliferation/migration by inducing ubiquitination^19,33,34^, but the detailed mechanism is unknown. Thus, the effect of SiO_2_ on circHECTD1 expression was measured in MML1 cells. First, the amplification of circHECTD1 from the cDNA, but not the genomic DNA (gDNA), was confirmed using divergent primers (Fig. 3a). The sequence of circHECTD1 is shown in Figure S3 and Table S2. SiO_2_ induced a rapid increase in circHECTD1 expression in MML1 cells (Fig. 3b), which was confirmed using FISH (Fig. 3c). Moreover, SiO_2_ also induced circHECTD1 expression in human endothelial cell line-HUVECs (Figure S4).

### circHECTD1 mediates the SiO_2_-induced EndMT

**Fig. 4:**
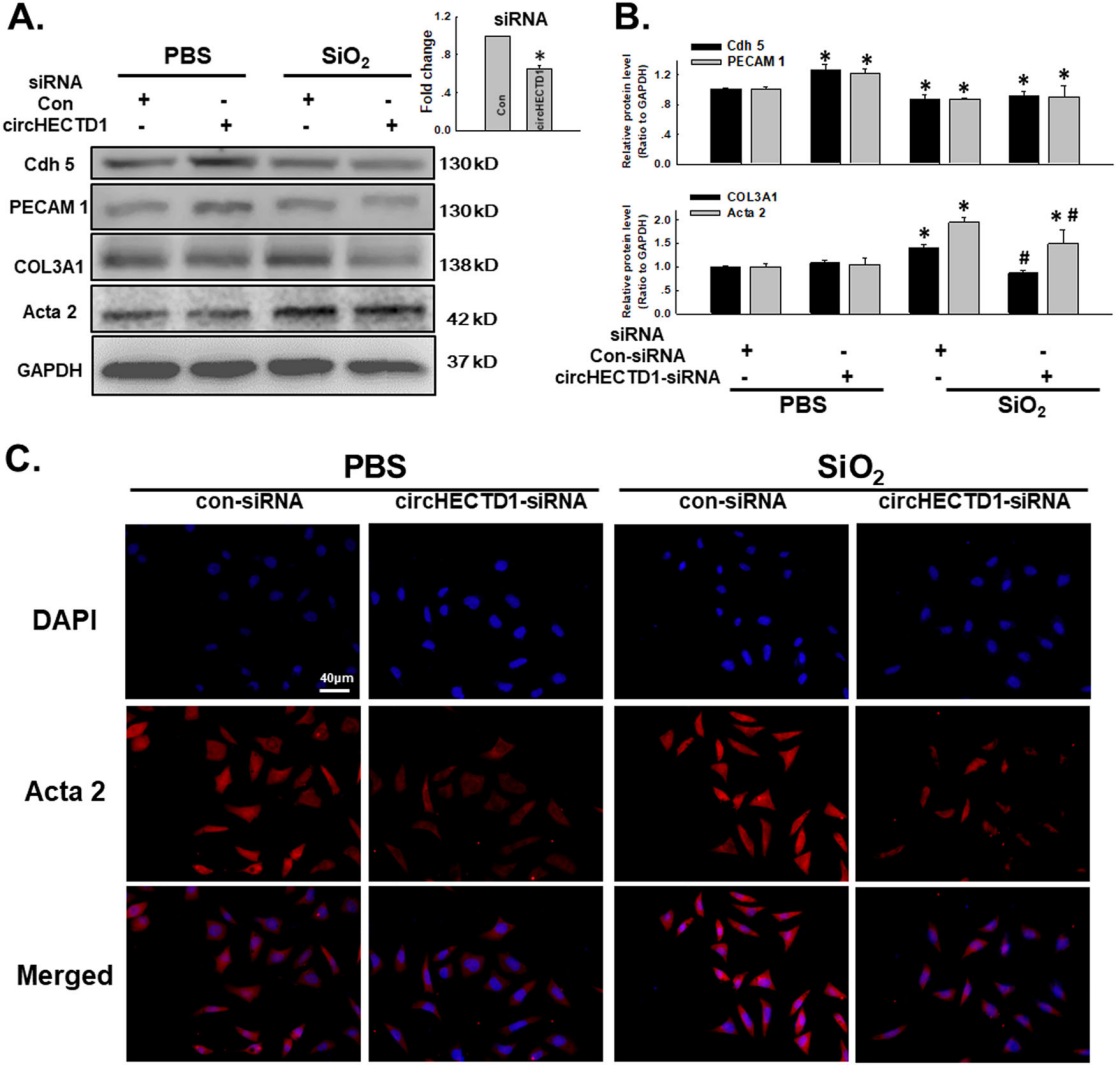
circHECTD1 mediates the SiO_2_-induced EndMT. **a** Representative western blot showing the effect of specific knockdown of circHECTD1 with siRNAs on SiO_2_-induced changes in endothelial and mesenchymal marker expression. The small pane shows the efficacy of circHECTD1-siRNA in reducing circHECTD1 expression. *P < 0.05 vs. the control group, *n* = 5. **b** Densitometric analyses of five separate experiments suggest that the SiO_2_-induced changes in the levels of endothelial and mesenchymal markers were attenuated by the circHECTD1-siRNA. *P < 0.05 vs. the level of the corresponding protein in the control group; #*P* < 0.05 vs. the level of the corresponding protein in the SiO_2_ group. **c** Representative images of immunocytochemical staining show that SiO_2_-induced α-SMA/Acta2 expression was attenuated by circHECTD1-siRNA

**Fig. 5:**
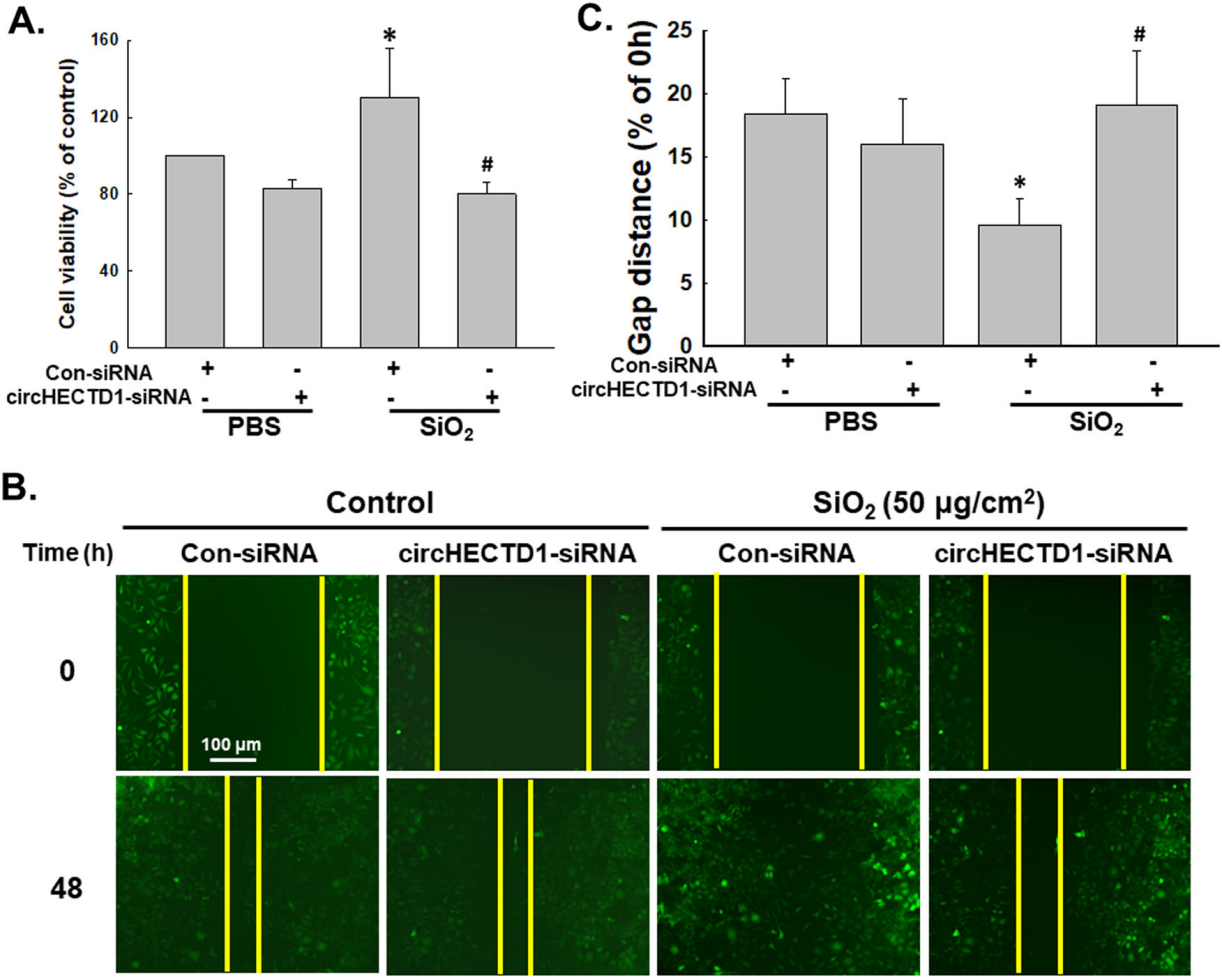
circHECTD1 is involved in SiO_2_-induced MML1 cell proliferation and migration. **a** MTT assay showing that circHECTD1-siRNA attenuates the SiO_2_-induced increase in MML1 cell viability. *P < 0.05 vs. the control group; #*P* < 0.05 vs. the SiO_2_ group, *n* = 5. **b** Representative images show that the effect of SiO_2_ on GFP-labeled MML1 cell migration in scratch assays was attenuated by circHECTD1-siRNA. Scale bar = 80 µm. **c** Quantification of the scratch gap distances in six separate experiments. *P < 0.05 vs. the control group; #*P* < 0.05 vs. the SiO_2_ group

Because SiO_2_ affected circHECTD1 expression in endothelial cells, we aimed to clarify whether circHECTD1 was involved in the SiO_2_-induced EndMT. As shown in Fig. 4a, b, specific knockdown of circHECTD1 using siRNAs significantly inhibited the SiO_2_-induced decreases in VE-Cad/Cdh-5 and PECAM 1/CD31 expression and the increases in Col III/COL3A1 and α-SMA/Acta2 expression, which were confirmed by immunostaining (Fig. 4c). Meanwhile, the SiO_2_-induced increases in endothelial cell viability and migration were also significantly reversed after circHECTD1 knockdown (Fig. 5a, c).

### circHECTD1 is involved in SiO_2_-induced HECTD1 downregulation

**Fig. 6:**
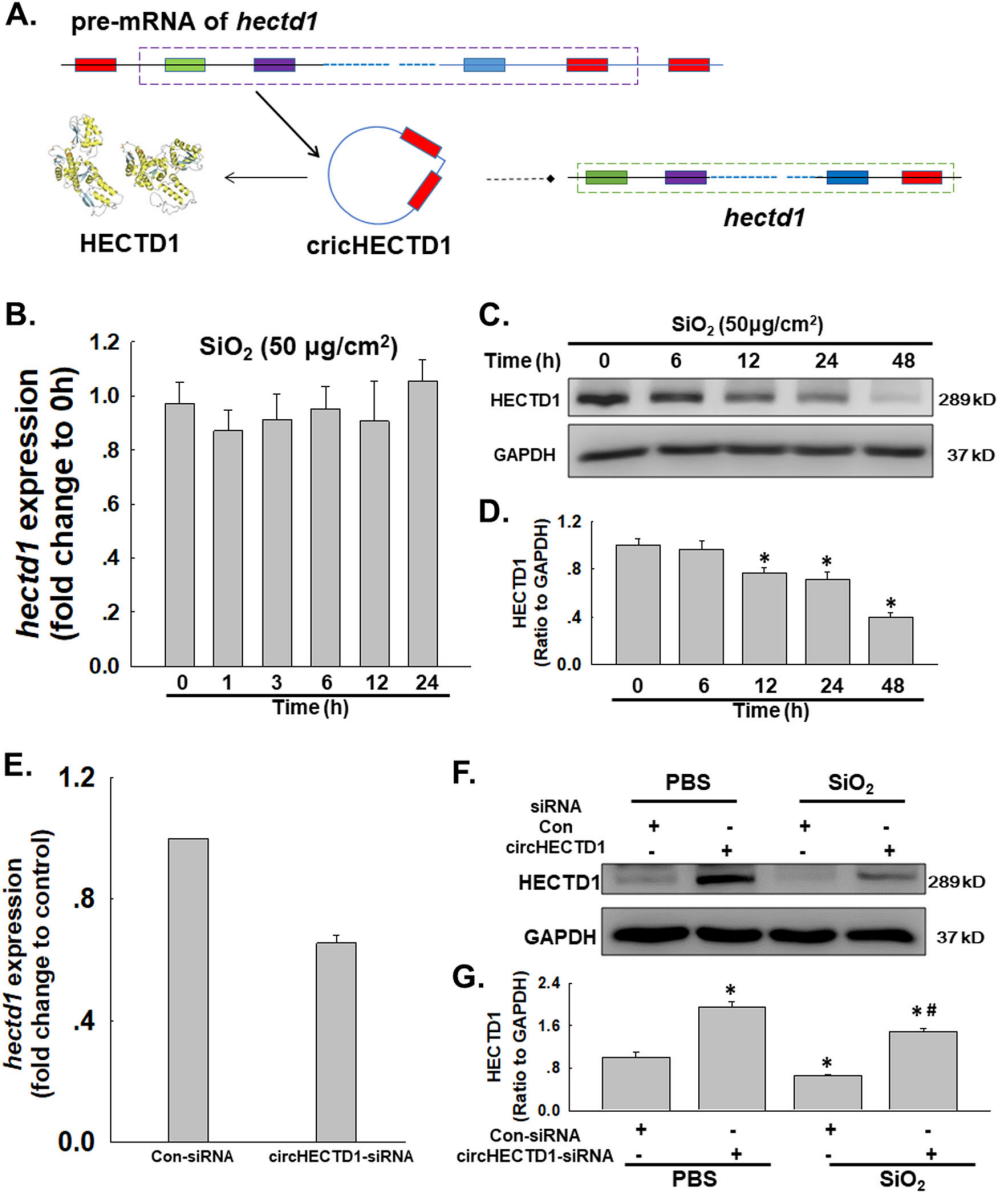
circHECTD1 is involved in SiO_2_-induced HECTD1 downregulation. **a** In the SiO_2_-induced EndMT, circHECTD1 may regulate the expression of its host gene, *hectd1*. **b** As shown in the qRT-PCR analysis, levels of the *hectd1* mRNA did not change in MML1 cells exposed to SiO_2_ (*n* = 5). **c** Representative western blot showing the effect of SiO_2_ on the level of the HECTD1 protein. **d** Densitometric analyses of five separate experiments suggest that SiO_2_ increases the level of the HECTD1 protein in MML1 cells. *P < 0.05 vs. protein expression at 0 h. **e** As shown in the qRT-PCR analysis, the expression of the *hectd1* mRNA decreased in MML1 cells treated with circHECTD1-siRNA. *P < 0.05 vs. the control group, *n* = 5. **f** Representative western blot showing the effect of specific knockdown of circHECTD1 with siRNAs on SiO_2_-induced HECTD1 expression. **g** Densitometric analyses of five separate experiments suggest that SiO_2_-induced changes in HECTD1 expression are attenuated by circHECTD1-siRNA. *P < 0.05 vs. the levels of the corresponding protein in the control group; #*P* < 0.05 vs. the levels of the corresponding protein in the SiO_2_ group

After determining the role of circHECTD1 in the SiO_2_-induced EndMT, we investigated the involvement of its host gene, HECTD1 (Fig. 6a). As shown in Fig. 6b–d, the expression of the *hectd* mRNA in MML1 cells was not altered after SiO_2_ exposure, whereas the level of the HECTD1 protein decreased in a time-dependent manner. Moreover, although specific knockdown of circHECTD1 induced a decreasing trend in the expression of the *hectd1* mRNA, the circHECTD1-siRNA not only increased the level of the HECTD protein in the normal MML1 cells but also restored the HECTD1 level in MML1 cells exposed to SiO_2_ (Fig. 6e–g), indicating a role for HECTD1 in the SiO_2_-induced EndMT.

### HECTD1 is involved the SiO_2_-induced EndMT in MML1 cells

**Fig. 7:**
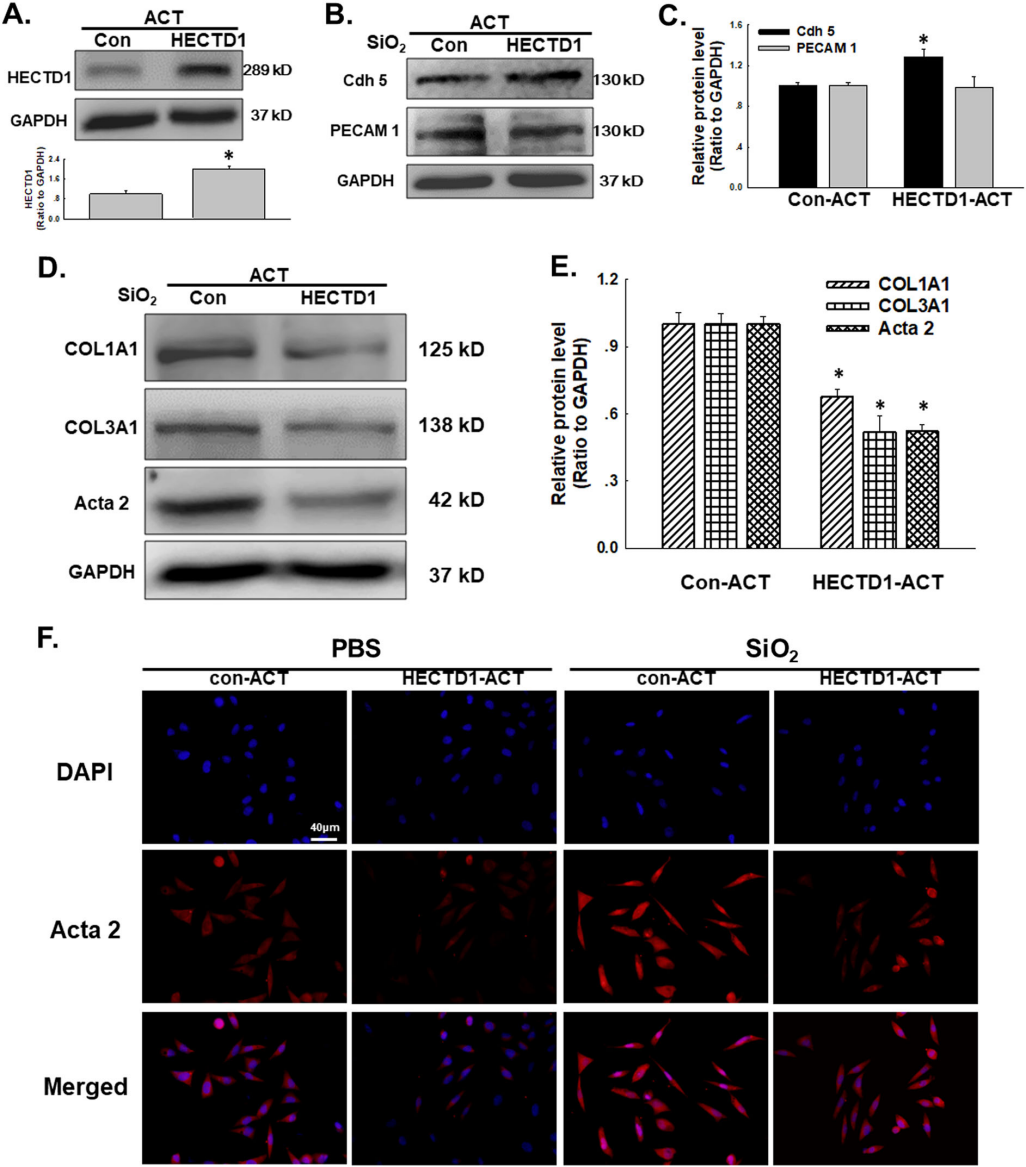
HECTD1 is involved in the SiO2-induced EndMT in MML1 cells. **a** Representative western blot and densitometric analyses showing the efficacy of HECTD1 CRISPR ACT at increasing the level of the HECTD1 protein. *P < 0.05 vs. the control group, *n* = 5. **b** Representative western blot showing the effect of specific upregulation of HECTD1 expression with ACT on SiO_2_-induced endothelial marker expression. **c** Densitometric analyses of five separate experiments suggest that the SiO_2_-induced changes in VE-Cad/Cdh-5 levels, but not PECAM 1/CD31 levels, are attenuated by HECTD1 ACT. *P < 0.05 vs. the level of the corresponding protein in the control group; #*P* < 0.05 vs. the level of the corresponding protein in the SiO_2_ group. **d** Representative western blot showing the effect of specific upregulation of HECTD1 with ACT on SiO_2_-induced mesenchymal marker expression. **e** Densitometric analyses of five separate experiments suggest that the SiO_2_-induced changes in mesenchymal markers are attenuated by HECTD1 ACT. *P < 0.05 vs. the level of the corresponding protein in the control group; #*P* < 0.05 vs. the level of the corresponding protein in the SiO_2_ group. **f** Representative images of immunocytochemical staining show that SiO_2_-induced α-SMA/Acta2 expression was attenuated by HECTD1 ACT

**Fig. 8:**
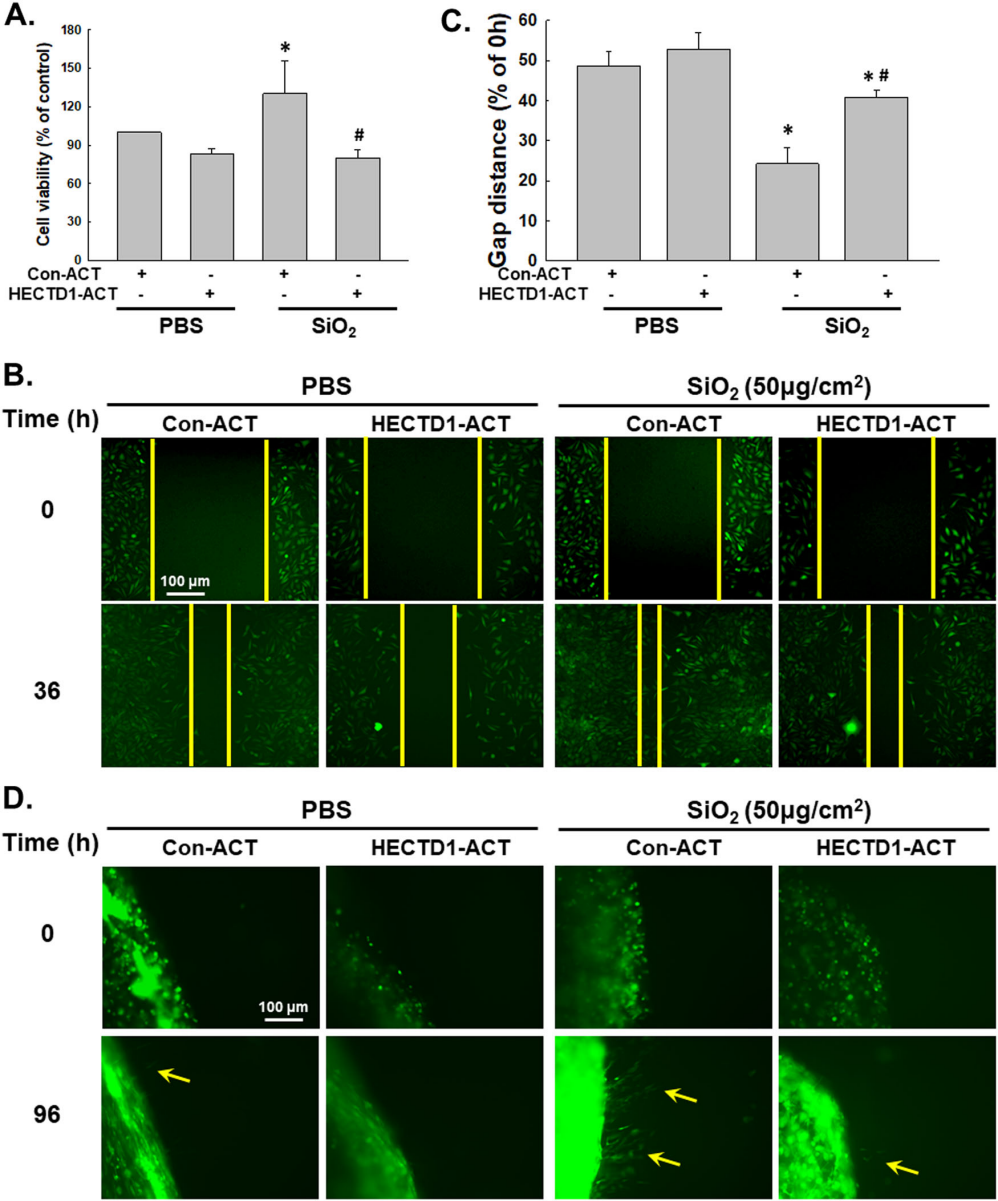
HECTD1 is involved in SiO_2_-induced MML1 cell proliferation and migration. **a** MTT assay showing that HECTD1 ACT attenuated the SiO_2_-induced increase in MML1 cell viability. *P < 0.05 vs. the control group; #*P* < 0.05 vs. the SiO_2_ group, *n* = 5. **b** Representative images show that the effect of SiO_2_ on GFP-labeled MML1 cell migration in scratch assays was attenuated by HECTD1 ACT. Scale bar = 80 µm. **c** Quantification of the scratch gap distances in six separate experiments. *P < 0.05 vs. the control group; #*P* < 0.05 vs. the SiO_2_ group. **d** Representative images show that the effect of SiO_2_ on GFP-labeled MML1 cell migration in the nested matrix cell migration assay was attenuated by HECTD1 ACT. Scale bar = 80 µm

Functional experiments were performed to investigate the effects of HECTD1 on cell proliferation and migration and to obtain a better understanding of the role of HECTD1 in the SiO_2_-induced EndMT. As shown in Fig. 7a, the HECTD1-CRISPR activation plasmid (ACT) significantly increased the HECTD1 levels in MML1 cells. Meanwhile, upregulation of HECTD1 in the cells restored the SiO_2_-induced decrease in VE-Cad/Cdh-5 and PECAM 1/CD31 (Fig. 7b, c) levels and the SiO_2_-induced increase in Col I/COL1A1, Col III/COL3A1 and α-SMA/Acta2 levels (Fig. 7d, e). This effect was confirmed using immunostaining (Fig. 7f). Moreover, the increases in endothelial cell viability and migration induced by SiO_2_ were also significantly reversed by the HECTD-ACT treatment (Fig. 8a, c). Additionally, the nested matrix cell migration assay was performed to confirm the role of HECTD1 in cell migration. As shown in Fig. 8d, the SiO_2_-induced increase in cell migration was attenuated by the HECTD-ACT treatment, suggesting a role for HECTD1 in the SiO_2_-induced EndMT.

### HECTD1 is involved the EndMT in vivo

**Fig. 9:**
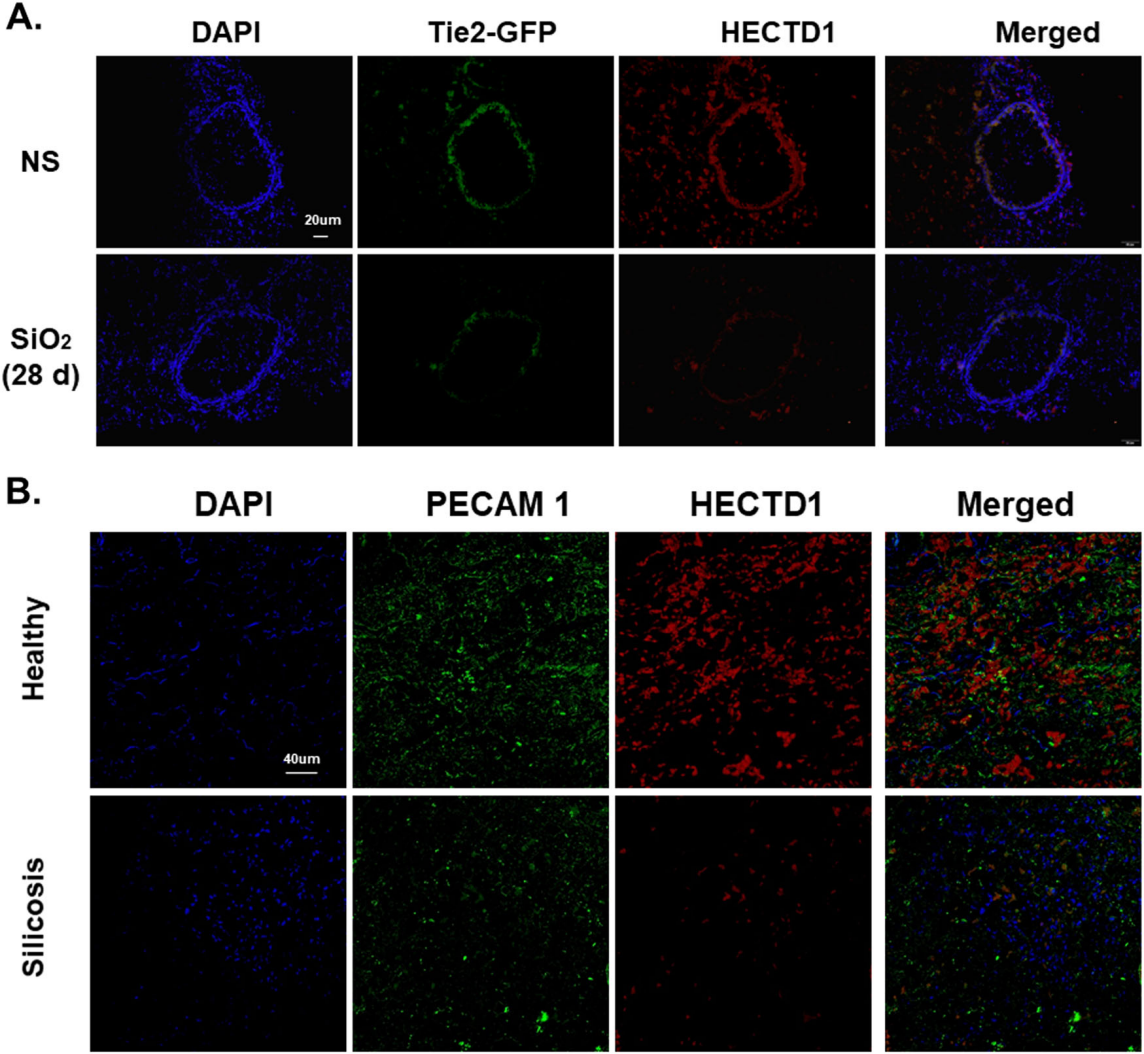
HECTD1 is involved in the EndMT in vivo. **a** Representative images of immunohistochemical staining show that Tie2-GFP co-localization with HECTD1 decreased in TEK-GFP 287 Sato/JNju mice treated with SiO_2_. **b** Representative images of immunohistochemical staining show the expression patterns of PECAM 1/CD31 and HECTD1 in lung sections from healthy donors and patients with silicosis

HECTD1 levels were evaluated in lung tissue samples from both mice and patients to validate our in vitro findings. As shown in Fig. 9a HECTD1 did not co-localize with GFP in normal mice after the SiO_2_ treatment. Lung tissues from the healthy donors and patients with silicosis showed decreases in PECAM 1/CD31 and HECTD1 staining, which supported our in vitro findings (Fig. 9b).

## Discussion

Pulmonary fibrosis is a key and late response of silicosis, and no effective therapies or drugs are currently available to prevent or minimize this progression^2,35^. In addition to fibroblasts, both epithelial cells and endothelial cells contribute to fibrosis via the EMT and EndMT, respectively, and the detailed mechanisms remain unclear^3–5^. Compared to the EMT, which has been widely studied, the EndMT has received little attention in fibrotic diseases. The current study mainly focused on the roles of circRNAs and their downstream effects on the SiO_2_-induced EndMT, revealing a new strategy for treating silicosis.

SiO_2_ destroys the barrier between the air and blood, which exposes endothelial cells to silica^36^. This disruption results in not only activation of endothelial cells but also the EndMT, which exacerbates endothelial cell dysfunction^1,4^. The EndMT is characterized by the loss of an endothelial phenotype, the acquisition of a mesenchymal phenotype, and functional changes in the cell, including proliferation and migration^1,9^. Similar to the EMT, the EndMT is induced by transforming growth factor (TGF-β)^9^. For example, endothelial cell-derived fibroblasts participate in bleomycin-induced lung fibrosis, which is attenuated following the withdrawal of TGF-β both in vitro and in vivo^4^. Interestingly, hypoxia, a common feature of many diseases, plays an important role in various fibrotic diseases, including SSc, kidney, cardiac, and pulmonary fibrosis^37^. For example, hypoxia-induced HIF-1α expression promotes the EndMT during the development of radiation-induced pulmonary fibrosis^38^, indicating a role for the lung as a source triggering the EndMT in subjects with fibrotic diseases. Despite the importance of the EndMT during experimentally induced pulmonary fibrosis^4^, no reports have described the EndMT in patients with idiopathic pulmonary fibrosis or other forms of secondary pulmonary fibrosis^37^; however, the current study has provided another example of the EndMT in patients with pulmonary fibrosis.

Although the mechanism underlying the EndMT in pulmonary fibrosis deserves further investigation, several studies of cardiac fibrosis associated with the EndMT have indicated a role for noncoding RNAs in this pathological process. For example, the EndMT observed in the hearts of wild type diabetic mice is prevented in miR-200b transgenic diabetic mice, suggesting that miR-200b exerts a protective effect by inhibiting TGF-β1 and p300 expression^11^. Interestingly, miR-200a negatively regulates levels of the GRB2 protein by directly binding to the *grb2* 3′UTR, which, in turn, inhibits the EndMT in a cardiac interstitial fibrosis model^39^. Unlike miRNAs, which have been widely studied, circRNAs comprise a newly identified and highly abundant RNA species that has received increasing attention in recent decades. The circular structure of circRNAs is associated with high biochemical stability and conservation^40^, making circRNAs ideal candidate biomarkers for the diagnosis and targeted treatment of diseases^41^. Recent studies have suggested the potential use of circRNAs in the diagnosis and treatment of nervous system disorders and cancer^16,42–44^. For example, circHIPK2 promotes astrocyte and fibroblast activation through a ceRNA-mediated mechanism, in which circHIPK2 functions as an endogenous miRNA sponge, resulting in increased SIGMAR1 expression^16,45^. Based on our previous circRNA microarray data and EndMT studies^32^, we selected circHECTD1, which promoted the EndMT in vitro after SiO_2_ exposure, in the current study. Unlike the classical ceRNA mechanism, circHECTD1 may regulate the protein level of its host gene, *hectd1*, through competition with its pre-mRNA, although further experiments are needed to confirm this hypothesis. The involvement of circHECTD1 in the SiO_2_-induced EndMT highlights circRNAs as new potential targets for silicosis treatment.

Although the detailed mechanism underlying the effects of circHECTD1 on the EndMT requires further investigation, circHECTD1 promotes functional changes in cells through HECTD1. HECTD1 is an E3 ubiquitin ligase that contains an N-terminal ankyrin repeat, a MIB domain, and a C-terminal HECT domain, which determines target protein specificity^46^; HECTD1 plays an important role in the ubiquitin-proteasome system^47^. HECTD1 is required for developmental processes in tissues, such as head mesenchyme and neural tube closure^48^ and placental junctional zone formation^49^, which are associated with the role of HECTD1 in cell migration^50,51^. Wnt signaling mediated by APC-Axin interactions is involved in regulating HECTD1 function^46^. Accordingly, excessive cell migration and proliferation are the obvious feature of the fibrosis process, which has led us to clarify the role of HECTD1 in EndMT-associated fibrosis in patients with silicosis. Moreover, a previous finding of the involvement of another important protein involved in ubiquitination, MCPIP1/ZC3H12A, strongly suggests a role for HECTD1 in silicosis^1^. The current study has suggested a negative regulatory role for HECTD1 in the EndMT through its ability to inhibit cell viability and migration, consistent with a previous finding showing that HECTD1 inhibits cell migration via Hsp90^50^. However, the downstream effects of HECTD1 after SiO_2_ exposure deserve further investigation. Additionally, our analyses of HECTD1 expression in patients with silicosis showed that HECTD1 expression was decreased, consistent with our in vitro results, thus confirming the clinical significance of our findings and revealing that HECTD1 may serve as a potential marker of silicosis.

## Conclusion

**Fig. 10:**
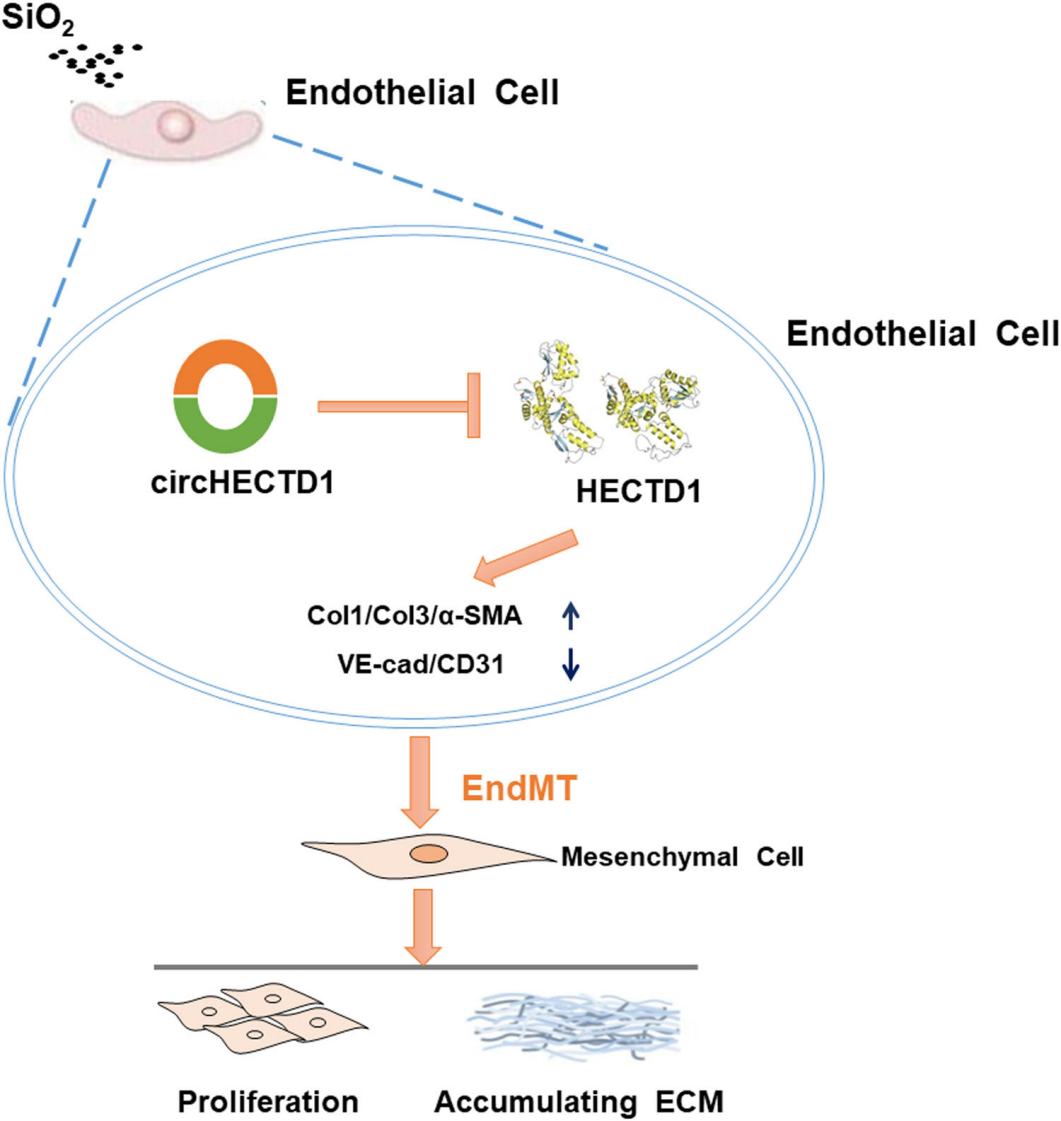
Schematic diagram showing the mechanisms by which circHECTD1/HECTD1 regulate the SiO_2_-induced EndMT. circHECTD1 expression is increased in endothelial cells exposed to SiO_2_. The increased expression leads to a subsequent decrease in HECTD1 expression, which, in turn, increases the levels of mesenchymal markers and decreases the levels of endothelial markers. Endothelial cell proliferation and migration are triggered, thereby contributing to irreversible fibrosis in patients with silicosis

Our study has elucidated a link between the SiO_2_-induced EndMT and the circHECTD1/HECTD1 pathway, thereby providing insights into the potential use of HECTD1 for developing novel therapeutic strategies for silicosis (Fig. 10).

### Availability of data and materials

All relevant raw data and materials are freely available to any scientist upon request.

## Electronic supplementary material

Supplemental data(DOCX 878 kb)
