# Supplementary material for: circHECTD1 promotes the silica-induced pulmonary endothelial–mesenchymal transition via HECTD1
Source: Cell Death Dis. 2018 Mar 14;9(3):396. doi: 10.1038/s41419-018-0432-1 (PMC5852113; doi:10.1038/s41419-018-0432-1)
Supplement: Supplementary file 1 — Supplemental data(DOCX 878 kb) [file 41419_2018_432_MOESM1_ESM.docx]

**Supplementary information**

**Manuscript title: circHECTD1 promotes the silica-induced pulmonary endothelial-mesenchymal transition *via* HECTD1**

Authors: Fang, Guo, Cheng, Zhou, Zhang, Han, Luo, Wang, Xie and Chao

**Table of contents**

Supplementary Figure S1 2

Supplementary Figure S2 3

Supplementary Figure S3 4

Supplementary Figure S4 5

Supplementary Table S1 6

Supplementary Table S2 7

**Supplementary Figure S1**

**
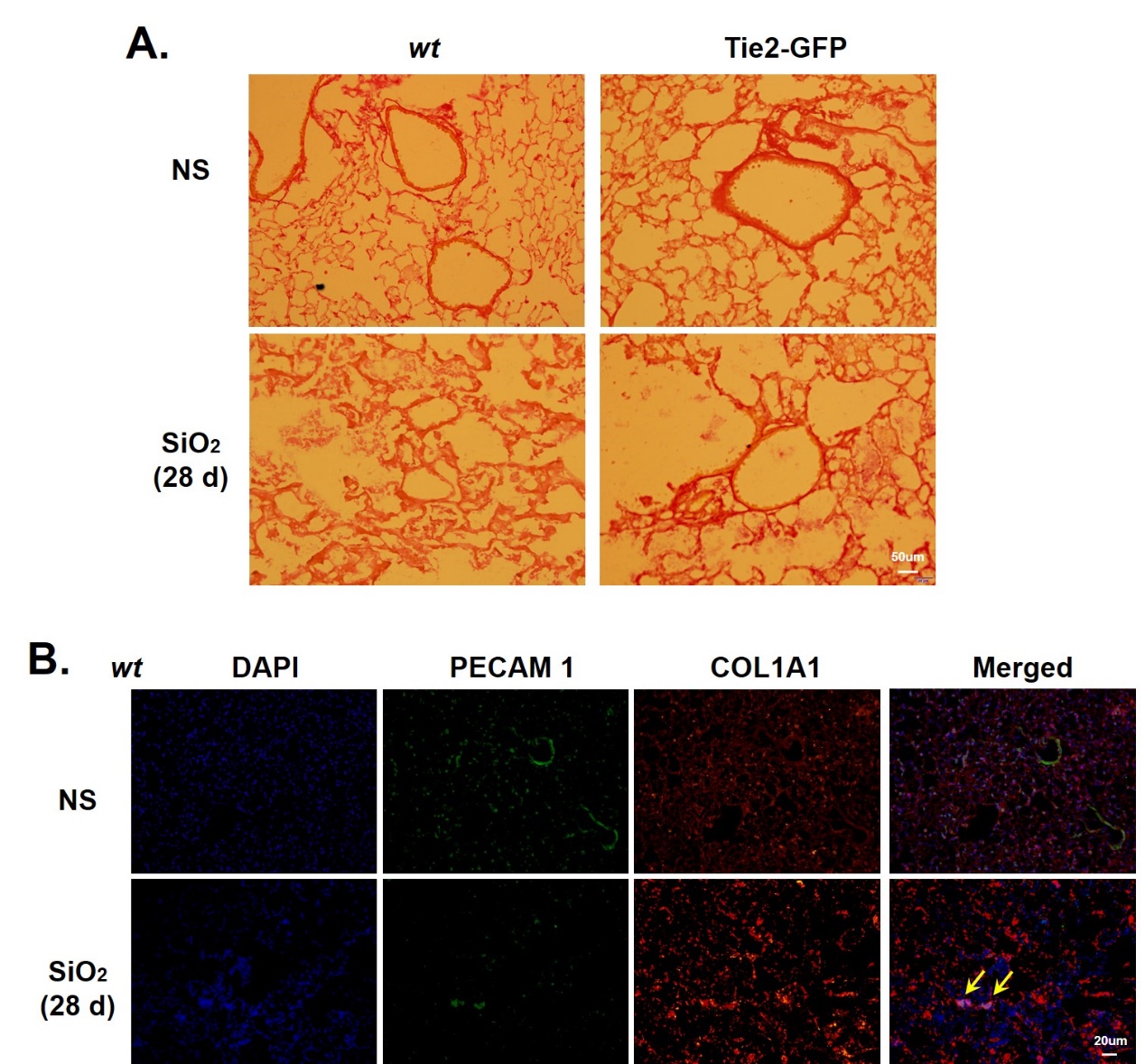
**

**Figure S1. SiO_2_ induces the EndMT**

**a.** Representative images of Sirius red staining in lung sections reflect collagen localization in wild type and Tie2-GFP mice treated with SiO_2_. **b.** Representative images of immunohistochemical staining show the expression patterns of PECAM 1/CD31 and COL1A1/Collagen I in lung sections from wild type mice.

**Supplementary Figure S2**


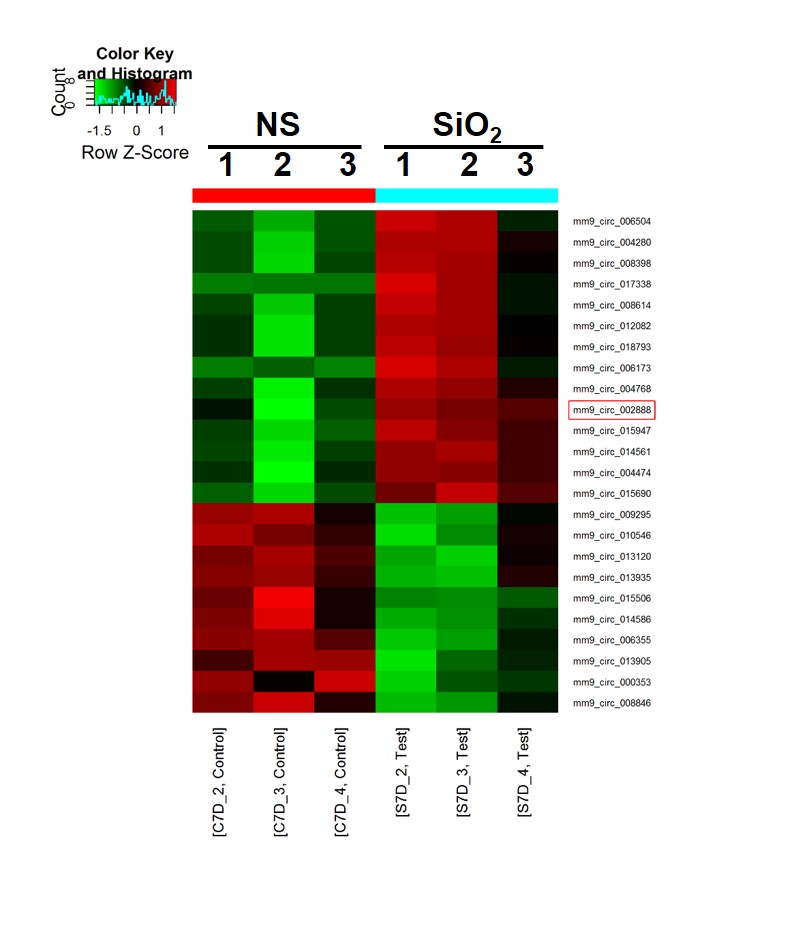


**Figure S2. Differential expression of circRNAs in mouse lung tissues**

Hierarchical clustering analysis of differentially expressed circRNAs in lung tissues from mice treated with NS and SiO_2_ (0.5 mg each); each group contained three animals (greater than a 2.5-fold difference in expression; *P*<0.05). Expression levels are presented in different colors indicating levels greater than and less than the median expression level across all samples.

**Supplementary Figure S3**

**
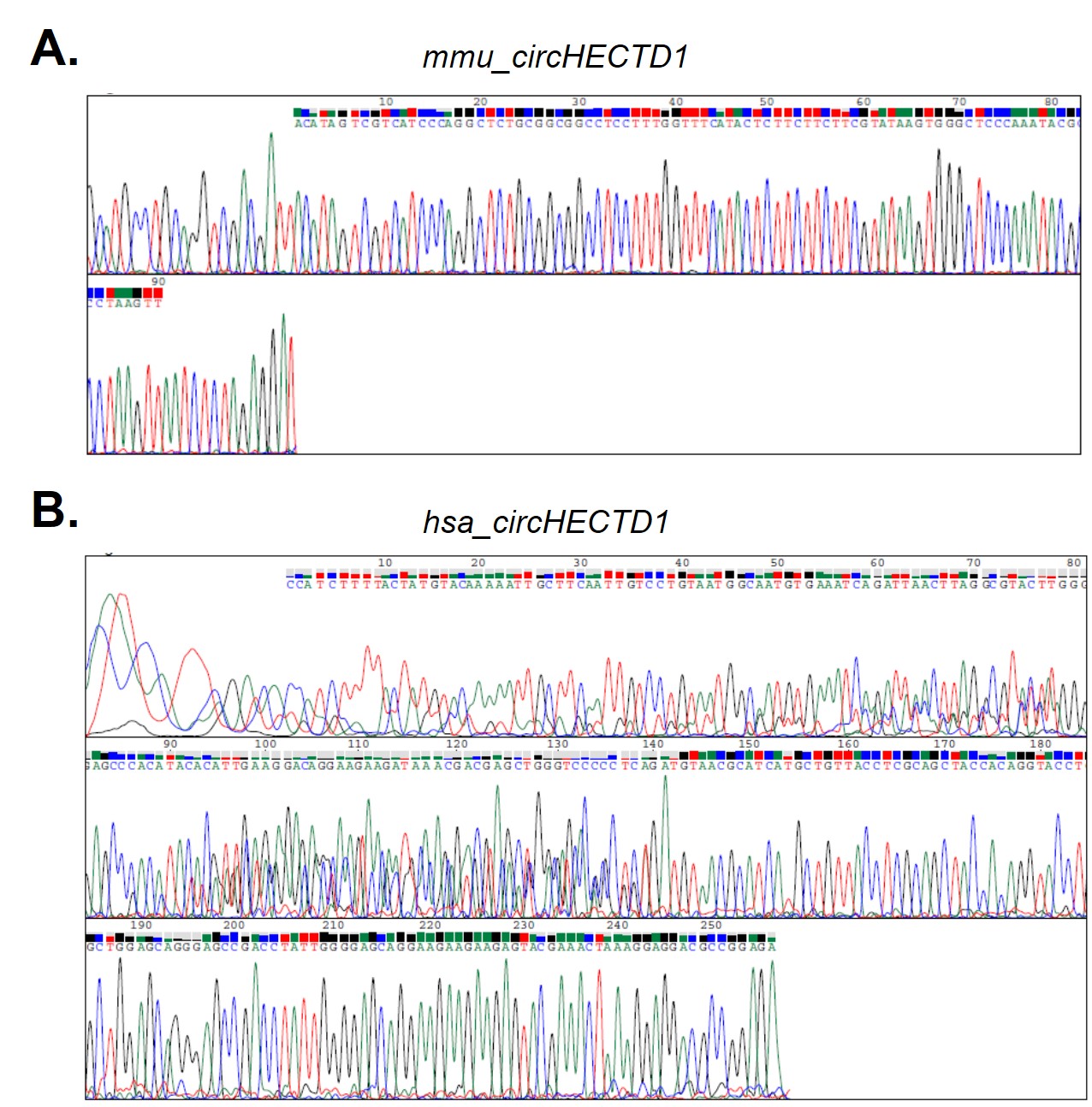
**

**Figure S3. The PCR-Sequencing results for the circHECTD1 gene.**

**a.** Direct sequencing chromatograms of mmu_circHECTD1 PCR product. **b.** Direct sequencing chromatograms of hsa_circHECTD1 PCR product..

**Supplementary Figure S4**

**
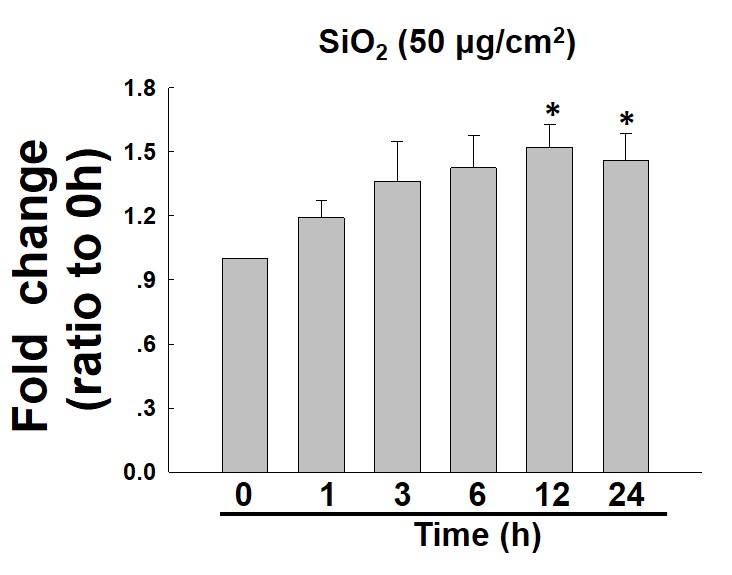
**

**Figure S4. SiO_2_ induces circHECTD1 expression in HUVECs**

As shown in the qRT-PCR analysis, circHECTD1 expression increased in HUVEC cells exposed to SiO_2_ (n=5). **P<*0.05 vs*.* circHECTD1 expression at 0 h.

**Table S1. Sequences of qRT-PCR primers and FISH probe**

| **mRNA qPCR primers** | | | |
| --- | --- | --- | --- |
| **Gene** | **Forward (5′-3′)** | | **Reverse (5′-3′)** |
| ***gapdh* (mouse)** | **TGTGTCCGTCGTGGATCTGA** | | **CCTGCTTCACCACCTTCTTGA** |
| ***hectd1* (mouse)** | **TTGAAACATGTCCACCTCGT** | | **CACGGGCTGTCACCTCTAAG** |
| **circRNA qPCR primers** | | | |
| **Name** | **Forward (5′-3′)** | | **Reverse (5′-3′)** |
| **mmu_circHECTD1 (Divergent primer)** | **AACTTAGGCGTATTTGGGAGC** | | **ACATAGTCGTCATCCCAGGC** |
| **mmu_circHECTD1 (Convergent primer)** | **GCCTGGGATGACGACTATGT** | | **GCTCCCAAATACGCCTAAGTT** |
| **has_ circHECTD1 (Divergent primer)** | **ACTCCGTCACCTCGATTAGC** | | **ATCATCCCATGTTCTCCGGC** |
| **hsa_circHECTD1 (Convergent primer)** | **GCCGGAGAACATGGGATGAT** | | **GCTAATCGAGGTGACGGAGT** |
| **FISH probe** | | | |
| **circRNA** | | **Sequence** | |
| **mmu_circHECTD1 (Biotin-labeled FISH probe)** | | **aaaCATACTCTTCTTCTTCGTGTAAGTGGGCTCCC** | |

**Table S2. Sequence of circHECTD1**

| **circRNA** | **Sequence** |
| --- | --- |
| **mmu_circHECTD1** | **GAAGAAGAAGAGTATGAAACCAAAGGAGGCCGCCGCAGAGCCTGGGATGACGACTATGTGCTAAAGCGCCAGTTTTCTGCACTGGTCCCTGCTTTTGATCCTAGACCTGGTCGTACCAATGTCCAGCAGACAACTGACCTAGAAATTCCTCCCCCAGGAACACCTCACTCAGAGCTCTTGGAGGAAGTTGAATGTACTCCGTCACCTCGCTTGGCTCTCACACTGAAAGTGACGGGGCTTGGAACAACGCGGGAAGTTGAACTGCCACTTACCAATTTCAGATCCACCATCTTTTACTATGTACAAAAACTGCTTCAACTGTCTTGTAATGGCAATGTGAAGTCAGATAAACTTAGGCGTATTTGGGAGCCCACTTACAC** |
| **hsa_circHECTD1** | **GAAGAAGAAGAGTACGAAACTAAAGGAGGACGCCGGAGAACATGGGATGATGATTATGTGCTAAAGAGACAGTTTTCTGCATTGGTTCCTGCTTTTGATCCTAGACCTGGTCGTACTAATGTCCAGCAGACAACTGATCTAGAAATACCACCCCCAGGGACCCCTCATTCAGAGCTCTTGGAAGAAGTCGAATGTACTCCGTCACCTCGATTAGCTCTCACTTTGAAAGTAACAGGTCTTGGAACGACTCGTGAAGTTGAATTACCACTCACCAATTTCAGATCAACCATCTTTTACTATGTACAAAAATTGCTTCAATTGTCCTGTAATGGCAATGTGAAATCAGATAAACTTAGGCGTATTTGGGAGCCCACATACAC** |
